# Real-world comparative effectiveness of sarilumab versus Janus kinase inhibitors as monotherapy in rheumatoid arthritis

**DOI:** 10.1186/s13075-025-03722-5

**Published:** 2026-01-02

**Authors:** Yuji Nozaki, Kazuya Kishimoto, Tetsu Itami, Daisuke Tomita, Yumiko Wada, Takuya Kotani, Tohru Takeuchi, Toshihiko Hidaka, Shoichi Hino, Toshiaki Miyamoto, Hirofumi Miyake, Kazunari Hatta, Kenji Mamoto, Yutaro Yamada, Tadashi Okano, Takaichi Okano, Jun Saegusa, Masahiro Horita, Keiichiro Nishida, Koji Kinoshita, Shinya Rai

**Affiliations:** 1 Department of Hematology and Rheumatology, Kindai University Faculty of Medicine, Osaka-sayama, Osaka, 589-8511 Japan; 2 Department of Internal Medicine (IV), Osaka Medical and Pharmaceutical University, Takatsuki, Japan; 3 Rheumatology Center, Miyazaki Zenjinkai Hospital, Miyazaki, Japan; 4 Department of Rheumatology and Clinical Immunology, Izumi City General Medical Center, Osaka, Japan; 5 Miyamoto Internal Medicine and Rheumatology Clinic, Hamamatsu, Japan; 6 Seirei Hamamatsu General Hospital, Hamamatsu, Japan; 7 Department of General Internal Medicine, Tenri Hospital, Nara, Japan; 8 Department of Orthopaedic Surgery, Graduate School of Medicine, Osaka Metropolitan University, Osaka, Japan; 9 Center for Senile Degenerative Disorders (CSDD), Osaka Metropolitan University Graduate School of Medicine, Osaka, Japan; 10 Department of Rheumatology and Clinical Immunology, Kobe University Graduate School of Medicine, Kobe, Japan; 11 Department of Orthopaedic Surgery, Faculty of Medical Development Field, Okayama University, Okayama, Japan; 12 Locomotive Pain Center, Faculty of Medical Development Field, Okayama University, Okayama, Japan; 13 Centre for Lymphoid Cancer, British Columbia Cancer, Vancouver, BC Canada

**Keywords:** Rheumatoid arthritis, Methotrexate, Biological DMARDs

## Abstract

**Background:**

Sarilumab (SAR), an interleukin-6 receptor inhibitor (IL-6Ri), and Janus kinase inhibitors (JAKi) are approved options for rheumatoid arthritis (RA) when methotrexate (MTX) cannot be used. Real-world evidence for MTX-free monotherapy remains limited.

**Methods:**

We conducted a multicenter retrospective cohort study of RA patients receiving SAR or JAKi as MTX-free monotherapy. To reduce confounding, 1:1 propensity score matching was performed in the overall cohort (*n* = 252, 126 per group) and separately within treatment-line strata: Phase 2 first-line biologic/targeted synthetic disease-modifying antirheumatic drugs (b/tsDMARDs: 45 per group), Phase 3 second-line b/tsDMARDs (53 per group), and Phase 3 **≥** third-line b/tsDMARDs (47 per group). Outcomes over 12 months included drug retention, change in Clinical Disease Activity Index (CDAI), glucocorticoid (GC) tapering and discontinuation, low disease activity (LDA, CDAI ≤ 10), and safety profiles. Predictors of LDA were evaluated with logistic regression. This multicenter real-world.

**Results:**

Across matched strata by prior b/tsDMARDs, retention and CDAI change did not differ significantly between SAR and JAKi through 12 months. When classified by cause, adverse events (AEs)-related discontinuation was higher with JAKi, yielding lower AE-specific retention. Both groups demonstrated GC sparing overtime, with a greater increase in GC discontinuation for SAR than for JAKi in Phase 2. Baseline predictors of achieving LDA at 12 months included higher C-reactive protein (CRP) and platelet count (Plt) in both groups, with additional associations of younger age and lower hemoglobin (Hb) in the SAR. In safety analyses, overall AEs were less frequent with SAR than with JAKi, driven by lower risks of infection including herpes zoster, while other categories were similarly infrequent.

**Conclusion:**

SAR and JAKi showed no statistically significant differences in 12-month retention or disease control in MTX-free monotherapy settings. Higher CRP and Plt with lower Hb, particularly in younger patients, identified better response to SAR and support biomarker guided selection between IL-6Ri and JAKi. In Phase 2, GC discontinuation with SAR suggests a practical strategy to reduce AEs while maintaining efficacy. Prospective studies should validate these findings and define actionable thresholds.

**Supplementary Information:**

The online version contains supplementary material available at 10.1186/s13075-025-03722-5.

## Introduction

Rheumatoid arthritis (RA) is a chronic autoimmune inflammatory disease characterized by persistent synovitis, systemic inflammation, and progressive joint destruction. The therapeutic landscape of RA has advanced substantially with the introduction of biologic or targeted synthetic DMARDs (b/tsDMARDs) [1, 2], enabling more individualized treatment strategies based on disease activity, prognostic factors, and patient preferences.

Interleukin-6 (IL-6) plays a pivotal role in RA pathogenesis [3]. IL-6 receptor inhibitors (IL-6Ri), such as sarilumab (SAR), have demonstrated clinical efficacy both in combination with conventional synthetic DMARDs (csDMARDs) and monotherapy [4, 5]. Similarly, Janus kinase inhibitors (JAKi) show robust efficacy and are frequently prescribed for patients who are refractory to or intolerant of csDMARDs [6]. Methotrexate (MTX) remains the anchor csDMARD in RA, but in real-world practice a substantial proportion of patients cannot receive or continue MTX because of intolerance or contraindications, including older age, impaired renal function, pulmonary comorbidities such as RA-associated interstitial lung disease (RA-ILD), a history of MTX-associated lymphoproliferative disorder (MTX-LPD), and gastrointestinal disorders [7–9]. Although both IL-6Ri and JAKi are recommended as monotherapy options for patients unable to use MTX, real-world comparative data in MTX-free settings remain limited. Randomized controlled trials have demonstrated the efficacy of both classes as monotherapy in MTX-intolerant or MTX-naïve patients [10–12], but observational studies that evaluate these agents in MTX-free settings—incorporating detailed patient backgrounds, treatment histories, and longitudinal outcomes—are scarce. Moreover, to our knowledge, no published studies have directly compared predictors of treatment response between SAR and JAKi.

The objective of this study was to evaluate treatment retention and clinical disease activity—assessed using the Clinical Disease Activity Index (CDAI) [13]—and to examine glucocorticoid (GC)-sparing effects in patients with RA receiving MTX-free SAR or JAKi in a multicenter observational cohort. To minimize baseline imbalances, 1:1 propensity score matching (PSM) was performed in the overall cohort and separately within treatment-line strata—Phase 2 first-line, Phase 3 s-line, and Phase 3 third-line or later—to align patient characteristics. In addition, patients were stratified by prior exposure to b/tsDMARDs and by baseline biomarkers—including CDAI, C-reactive protein (CRP), white blood cell count (WBC), hemoglobin (Hb), platelet count (Plt), anti-citrullinated peptide antibody (ACPA), and rheumatoid factor (RF)—to identify predictors of treatment response to SAR and JAKi.

## Patients and methods

### Patients

This study used data from 13 facilities, including university-affiliated hospitals and specialized rheumatology centers: Kindai University Hospital; Osaka Medical and Pharmaceutical University Hospital; Miyazaki Zenjinkai Hospital; Izumi City General Medical Center; Miyamoto Internal Medicine and Rheumatology Clinic; Seirei Hamamatsu General Hospital; Tenri Hospital; Osaka Metropolitan University Hospital; the Center for Senile Degenerative Disorders at Osaka Metropolitan University; Kobe University Hospital; Kobe University Graduate School of Medicine; Okayama University Hospital; and the Musculoskeletal Pain Center at Okayama University Hospital.

RA was classified according to either the 1987 American College of Rheumatology (ACR) criteria or the 2010 ACR/European League Against Rheumatism (EULAR) criteria [14, 15]. During the study period, both frameworks were in routine use in Japan, with the 1987 criteria commonly applied in patients with long-standing disease established before or around 2010. Eligibility required meeting either the 1987 ACR or the 2010 ACR/EULAR criteria before the index date. We included patients who initiated treatment with either SAR (IL-6Ri monoclonal antibody) or a JAKi. In total, 260 SAR-treated and 212 JAKi-treated patients were analyzed. To minimize temporal bias and ensure both agents were available in the same treatment era, we included patients who initiated treatment between September 2017 (the date of sarilumab approval in Japan) and May 2024. The cohort included women across a broad age range, including women of childbearing potential; however, pregnancy intention was not systematically recorded. Treatment selection between SAR and JAKi was made at the discretion of the treating physicians in routine clinical practice, and we acknowledge that therapeutic choices may differ for women who are planning pregnancy.

### Safety outcomes

Adverse events (AEs) were recorded and categorized as infections, rash, hematologic, malignancy, RA-ILD (RA-associated interstitial lung disease) exacerbation, renal injury, and other events. We specifically identified AE discontinuations, defined as investigator-attributed AEs that resulted in permanent cessation of SAR or JAKi therapy. AE counts and risks (%) were summarized overall and by category. Analyses were performed in the overall cohorts after PSM (JAKi and SAR: *n* = 126).

### Study outcomes

Study outcomes were assessed over 12 months after initiation of SAR or JAKi. The primary outcome was 12-month drug retention, defined as continuation of the index SAR or JAKi without permanent discontinuation for any reason. Secondary outcomes included (1) change in CDAI (ΔCDAI) from baseline at 3, 6, and 12 months, (2) the proportions of patients achieving CDAI-low disease activity (LDA; CDAI ≤ 10.0) and remission (CDAI ≤ 2.8), (3) change in daily oral glucocorticoid (prednisolone-equivalent) dose (ΔGC) and cumulative GC discontinuation, and (4) safety profiles, including overall AEs, AE categories, and discontinuations due to AEs. Exploratory outcomes comprised phase-based retention and CDAI trajectories according to treatment line (Phase 2 first-line, Phase 3 s-line, and Phase 3 ≥ third-line b/tsDMARDs) and subgroup analyses stratified by prior b/tsDMARD exposure and baseline prognostic factors (CDAI, CRP, WBC, Hb, Plt, RF, ACPA, and age at initiation). Baseline predictors of achieving CDAI-LDA at 12 months were evaluated using logistic regression models within the matched cohort.

### Sample size and power considerations

The sample size of this study was determined by the number of eligible patients enrolled in the multicenter registry during the study period; no formal a priori sample size calculation was performed. After application of the inclusion and exclusion criteria and 1:1 propensity score matching, 252 patients (126 treated with SAR and 126 with JAKi) were available for comparative analyses. Given this fixed sample size, we performed a post-hoc assessment of the detectable effect size for our main outcomes (12-month drug retention and change in CDAI). Under conventional assumptions (two-sided α = 0.05) and the observed 12-month discontinuation rate of approximately 40% in the matched cohort, a cohort of 126 patients per group provides approximately 80% power to detect a hazard ratio of approximately 1.8 for 12-month drug discontinuation between groups. For the continuous outcome of ΔCDAI, the same sample size provides approximately 80% power to detect a between-group difference corresponding to an effect size of about 0.4 standard deviations. These calculations indicate that the study was powered to detect moderate or larger differences in treatment retention and disease activity, whereas smaller differences may not have been detectable.

### Statistical analysis

To reduce baseline imbalances between groups, 1:1 PSM was performed using nearest-neighbor matching without replacement and a caliper of 0.2 standard deviations of the logit of the propensity score, based on established methods [16, 17]. Variables included in the matching model were age, sex, disease duration, baseline CDAI, and concurrent use of GCs. Covariate balance before and after matching was assessed using standardized mean differences (SMDs), with SMD < 0.2 indicating sufficient balance. For transparency, SMDs for all covariates before and after matching are presented, and variables with small residual imbalances after PSM are explicitly indicated. To mitigate the potential influence of these residual imbalances, multivariable regression analyses were performed within the matched cohort, additionally adjusting for covariates that remained imbalanced after matching. Patients with missing key covariates were excluded. Treatment continuation was evaluated using Kaplan–Meier survival curves and compared with log-rank tests and Cox proportional hazards models. The proportional hazards assumption was assessed using Schoenfeld residuals. Changes in GC dosage were assessed at each time point using the Wilcoxon signed-rank test, while discontinuation rates were compared using chi-square tests. For analyses of predictors of treatment response, we used the PSM cohort described above (*n* = 252; 126 SAR and 126 JAKi). For GC outcomes, changes in oral glucocorticoid (prednisolone-equivalent) dose and cumulative GC discontinuation were analyzed on an observed-case basis. Patients contributed GC data up to the time of treatment discontinuation, and values after discontinuation were not imputed. In the main analysis, all patients in the PSM cohort were included, reflecting real-world prescribing patterns in which some patients initiate SAR or JAKi without concomitant GC. For the change in daily GC dose, patients not receiving GC at baseline contributed values of 0 mg/day, whereas for GC discontinuation, the denominator comprised all patients at risk and those not on GC at baseline were considered already off GC. Because GC doses were highly skewed with a substantial proportion of zero values, changes in GC dose were summarized as medians with interquartile ranges rather than means with standard deviations. Baseline predictors of achieving CDAI-LDA (CDAI ≤ 10.0) at 12 months were examined using multivariable logistic regression models within the matched cohort (as presented in Table 4), adjusted for treatment group and the covariates that showed residual imbalance after PSM. For CDAI, changes at 3, 6, and 12 months (ΔCDAI) were analyzed on an observed-case basis. Patients contributed CDAI data up to the time of treatment discontinuation, and CDAI values after discontinuation were not imputed. 12-month ΔCDAI was therefore calculated among patients with CDAI available at both baseline and 12 months. In addition to the primary comparison between SAR and JAKi in the matched cohort, we conducted stratified and quartile-based analyses according to prior b/tsDMARD use, baseline biomarker quartiles, and serological status. Because these subgroup analyses involved multiple comparisons and were primarily hypothesis-generating, no formal adjustment for multiple testing was applied, and the corresponding *p*-values are interpreted as exploratory. All analyses were conducted using JMP Pro 18.0 (SAS Institute, Cary, NC) and GraphPad Prism 10 (GraphPad Software, San Diego, CA).

## Results

### Patient characteristics

Of 472 eligible patients who initiated SAR (*n* = 260) or JAKi (*n* = 212), 40 (8.5%) were excluded from the propensity score analysis because of missing key baseline covariates required for propensity score estimation, leaving 432 patients with complete data. After 1:1 propensity score matching, 252 patients (126 SAR and 126 JAKi) were included in the matched cohort (Suppl. Table 2). Table 1 summarizes baseline characteristics before and after PSM, and values are reported as mean ± SD or median [IQR], as appropriate. We prespecified |SMD| < 0.10 as the balance target and did not perform hypothesis testing for baseline differences. Before matching, SAR (*n* = 260) vs. JAKi (*n* = 212) showed marked imbalance in treatment-line distribution, with 1 st vs. 2nd vs. ≥3rd line 47.7% vs. 26.5% vs. 25.8% in SAR vs. 24.5% vs. 32.1% vs. 43.4% in JAKi, and a higher inflammatory burden in the SAR group (CRP 1.8 vs. 0.6 mg/dL, ESR 53.0 vs. 29.0 mm/h). Hematologic indices were also higher with SAR (WBC 7995 vs. 7246/µL, neutrophils 5652.5 vs. 4891.4/µL, and PLt 29.1 vs. 25.8 × 10⁴/µL), while age and sex were comparable and disease activity was modestly higher with SAR (CDAI 22.0 ± 12.0 vs. 19.1 ± 10.4). Patients in the JAKi group also had a higher prevalence of RA-ILD (19.3% vs. 10.3%) and more frequent concomitant glucocorticoid use (52.7% vs. 39.8%), whereas renal function (eGFR and the proportion with eGFR ≤ 60 mL/min/1.73 m²) and the history of MTX-LPD were broadly comparable between groups. After 1:1 matching, the treatment-line distribution in SAR vs. JAKi was 25.4% vs. 26.2% for first-line, 35.7% vs. 34.1% for second-line, and 38.9% vs. 39.7% for ≥ third-line, and baseline characteristics were comparable, with age 68.6 ± 13.3 vs. 69.4 ± 12.1 years, female proportion 77.0% vs. 77.8%, and CDAI 20.7 ± 11.6 vs. 19.6 ± 9.0. Residual differences persisted mainly in inflammatory and patient-reported measures, with higher CRP and ESR and differences in HAQ-DI, while most other covariates were similar. In addition, modest residual differences remained in the prevalence of RA-ILD and in radiographic damage, with numerically higher proportions of Steinbrocker stage I and class 3–4 disease in the JAKi group. Tables 2 and 3 show the same pattern within strata of prior bDMARD exposure, with PSM equalizing demographics and most clinical measures across first-line, second-line, and ≥ third-line groups, while modest residual imbalances in inflammatory markers and selected patient-reported outcomes remained without compromising overall comparability of the matched cohorts. The composition of JAKi by agent is summarized in Table 1 for tofacitinib, baricitinib, peficitinib, upadacitinib, and filgotinib with percentages before and after matching. All five agents used during the study period were represented, and given small numbers in some post-match strata, drug-level findings are presented descriptively without hypothesis testing.

**Table 1:** Baseline clinical data, laboratory data, and SAR and JAKi treatment information of 472 rheumatoid arthritis patients

	Before propensity matching			After propensity matching		
	SAR: *n* = 260	JAKi: *n* = 212	SMD before matching	SAR: *n* = 126	JAKi: *n* = 126	SMD after matching
Age, years	69.4 ± 13.2	70.4 ± 11.5	0.08	68.6 ± 13.3	69.4 ± 12.1	0.06
Age ≥ 65 years	64.0	60.6	0.07	64.0	60.6	0.07
Female (%)	76.5	78.9	0.06	77.0	77.8	0.02
Disease duration, months	90.0 [15.5–202.5]	84.0 [30.0–187.0]	0.07	108.5 [32.5–192.0]	86.5 [26.8–194.3]	0.08
1st/2nd/≥3rd-line (%)	47.7/26.5/25.8	24.5/32.1/43.4	0.79	25.4/35.7/38.9	26.2/34.1/39.7	0.02
RF (%), titer (IU/mL)	79.5, 61.0 [16.0–196.0.0.0]	76.0, 45.3 [13.0–374.5.0.5]	0.08, 0.08	81.2, 59.0 [19.0–175.0.0.0]	75.2, 65.0 [13.0–421.0.0.0]	0.1, 0.1
ACPA (%), titer (IU/mL)	76.4, 46.2 [0.8–304.5.8.5]	70.4, 56.4 [0.4–305.0]	0.13	72.6, 48.1 [0.6–234.1.6.1]	68.3, 53.2 [0.3–233.0]	0.09, 0.09
CRP, mg/dL [IQR]	1.8 [0.5–4.9]	0.6 [0.1–2.1]	0.48	1.5 [0.2–4.7]	0.7 [0.1–2.0]	0.39
ESR, mm/hr [IQR]	53.0 [29.0–87.0]	29.0 [13.5–61.0]	0.45	51.0 [28.0–87.5.0.5]	28.0 [12.0–59.5.0.5]	0.28
Tender joints, range 0–28 [IQR]	4.0 [1.0–9.0]	3.0 [1.0–7.0]	0.19	4.0 [1.0–9.0]	3.0 [1.0–7.0]	0.22
Swollen joints, range 0–28 [IQR]	5.0 [2.0–8.0]	4.0 [1.0–6.0]	0.3	4.0 [2.0–7.0]	4.0 [2.0–6.0]	0.33
Patient visual analogue scale, 0–100 mm	55.0 [30.0–76.0]	52.0 [29.8–77.3]	0.16	50.0 [25.0–73.0]	58.0 [38.8–80.0]	0.35
Physician visual analogue scale, 0–100 mm	50.0 [30.0–70.0]	44.0 [26.0–69.0]	0.02	48.0 [27.5–65.0]	50.0 [30.0–72.3]	0.17
CDAI	22.0 ± 12.0	19.1 ± 10.4	0.25	20.7 ± 11.6	19.6 ± 9.0	0.1
HAQ-DI, range 0–3	1.0 [0.4–1.8]	0.8 [0.1–1.6]	0.20	1.0 [0.4–1.6]	1.4 [0.4–1.9]	0.25
WBC,/µL	7995.0 ± 2826.1	7246.0 ± 2688.9	0.27	7867.2 ± 2614.4	7192.4 ± 2678.5	0.18
Neut,/µL	5652.5 ± 2680.4	4891.4 ± 2298.8	0.29	5307.9 ± 238.5	4918.8 ± 222.1	0.16
Hb, g/dL	11.6 ± 1.7	11.8 ± 1.7	0.11	11.8 ± 0.2	11.8 ± 0.2	0.0
Plt, ×10⁴/µL	29.1 ± 11.4	25.8 ± 8.7	0.32	27.1 ± 9.4	25.3 ± 0.8	0.2
AST, IU/L	20.0 [16.0–25.0]	20.0 [17.0–27.0]	0.06	20.0 [16.0–25.5.0.5]	20.0 [17.0–26.0]	0.04
ALT, IU/L	13.0 [9.0–20.0]	14.0 [10.0–20.0]	0.06	14.0 [8.5–22.0]	14.5 [11.0–22.0]	0.09
Cr, mg/dL	0.9 ± 0.4	0.8 ± 0.3	0.0	0.9 ± 0.4	0.8 ± 0.5	0.04
eGFR (mL/min/1.73m^2^), eGFR ≦ 60	62.0 ± 24.5, 31.0	66.7 ± 23.8, 26.4	0.16, 0.10	64.2 ± 24.9, 32.4	63.9 ± 22.9, 32.8	0.0, 0.04
RA-ILD (%)	10.3	19.3	0.26	12.4	17.6	0.19
History of MTX LPD (%)	2.7	3.8	0.06	2.9	3.2	0.05
JAKi: TOF/BAR/PEF/UPA/FIL (%)		24.4/23.0/13.6/19.7/19.3			16.7/26.2/13.5/21.4/22.2-	
csDMARDs: SASP/IGU/BUC/TAC (%)	27.8/33.8/4.0/9.3	31.8/37.1/2.7/11.9		26.2/38.1/4.0/2.7/9.5	31.0/37.3/3.2/2.7/13.5	
Glucocorticoid (%), mg/day [IQR]	39.8, 0.0 [0.0–5.0]	52.7*, 1.0 [0.0–5.0]	0.25, 0.04	42.9, 0.0 [0.0–4.0]	49.8, 1.5 [0.0–5.0]	0.1, 0.1
Steinbrocker stage I/II/III/IV	32.2/22.0/21.2/24.6	40.2/18.4/17.8/23.6		30.3/24.4/21.9/22.5	49.1/17.9/14.2/18.9*	
Steinbrocker class 1/2/3/4	22.8/48.3/25.9/3.1	17.5/57.9/21.3/3.3		27.7/53.6/17.9/0.9	11.0/57.8/28.4/2.8*	

Values are median [interquartile range], unless otherwise indicated

*SAR* Sarilumab, *JAKi* JAK inhibitors, *SMD* standardized mean differences, *RF* rheumatoid factor, *ACPA* anticitrullinated peptide antibody, *CRP* C-reactive protein, *ESR* erythrocyte sedimentation rate, *CDAI* clinical disease activity index, *HAQ-DI* health assessment questionnaire disability index, *WBC* white blood cell, *Neut* neutrophil, *Hb* Hemoglobin, *Plt* Platelet count, *AST* aspartate aminotransferase, *ALT* alanine aminotransferase, *Cr* creatinine, *eGFR* estimated glomerular filtration rate, *TOF* Tofacitinib, *BAR *Baricitinib, *PEF* Peficitinib, *UPA* Upadacitinib, *FIL *Filgotinib, *csDMARDs* conventional synthetic disease-modifying antirheumatic drugs, *SASP* Salazosulfapyridine, *IGU* Iguratimod, *BUC* Bucillamine, *TAC* Tacrolimus

**Table 2:** Baseline clinical data, laboratory data, and SAR and JAKi treatment information of rheumatoid arthritis patients before propensity score matching

	Phase 2 (First-line b/tsDMARDs)			Phase 3 (Second-line b/tsDMARDs)			Phase 3 (≥ Third-line b/tsDMARDs)		
	SAR: *n* = 124	JAKi: *n* = 52	SMD	SAR: *n* = 69	JAKi: *n* = 68	SMD	SAR: *n* = 67	JAKi: *n* = 92	SMD
Age, years	71.8 ± 11.7	71.9 ± 13.4	0.01	67.4 ± 15.5	69.7 ± 11.0	0.17	66.8 ± 12.8	70.3 ± 11.1	0.29
Female (%)	73.4	76.9	0.08	76.8	75.0	0.04	82.1	82.6	0.01
Disease duration, months	19.0 [4.0–130.5]	26.0 [5.0–136.0]	0.05	95.0 [49.3–210.0]	59.0 [25.0–122.0]*	0.50	189.5 [124.5–301.0]	139.5 [70.3–231.8]	0.46
RF (%), titer (IU/mL)	72.8, 41.0 [10.3–196.0]	75.5, 47.5 [13.2–195.4.2.4]	0.06/0.022	85.7, 67.0 [18.0–197.0.0.0]	77.6, 44.0 [13.5–454.0]	0.21/0.31	85.0, 71.1 [32.3–240.8.3.8]	74.7, 70.0 [12.0–636.0.0.0]	0.26/0.36
ACPA (%), titer (IU/mL)	69.1, 30.6 [0.0–309.0.0.0]	60.5, 83.4 [0.3–322.6.3.6]	0.18/0.095	80.7, 100.0 [25.3–342.0]	71.7, 60.4 [0.5–424.5.5.5]	0.21/0.02	82.5, 52.9 [11.7–197.5.7.5]	74.7, 56.4 [0.7–199.8.7.8]	0.19/0.012
CRP, mg/dL [IQR]	3.0 [1.1–6.9]	1.3 [0.3–4.9]	0.39	1.0 [0.1–3.5]	0.4 [0.0–1.5]	0.46	1.1 [0.4–4.0]	0.3 [0.0–1.9]	0.52
ESR, mm/hr [IQR]	69.0 [39.0–93.0]	43.0 [21.0–74.5]	0.45	44.0 [19.0–67.8]	27.0 [6.8–58.0]	0.31	42.5 [27.3–71.0]	30.0 [12.0–59.0]	0.35
Tender joints, range 0–28 [IQR]	4.0 [1.0–10.0]	3.0 [1.0–8.0]	0.17	3.0 [1.0–6.0]	3.0 [1.0–10.0]	0.25	4.0 [2.0–10.0]	2.0 [1.0–5.5]	0.52
Swollen joints, range 0–28 [IQR]	5.0 [2.0–10.0]	4.0 [2.0–6.0]	0.35	3.0 [1.0–7.0]	4.0 [1.0–6.5]	0.04	5.0 [2.0–7.8]	3.0 [1.0–6.0]	0.40
Patient visual analogue scale, 0–100 mm	63.0 [39.0–79.0]	50.0 [30.0–75.3]	0.27	50.0 [22.8–70.3]	58.0 [30.5–78.0]	0.22	50.0 [26.0–73.8]	52.5 [22.8–78.0]	0.03
Physician visual analogue scale, 0–100 mm	61.5 [40.0–83.0]	43.0 [25.0–69.0]	0.49	44.0 [27.0–58.5]	55.0 [30.0–73.5]	0.34	45.0 [24.3–60.0]	39.0 [25.0–56.5]	0.12
CDAI	25.1 ± 13.4	18.5 ± 8.8	0.58	17.7 ± 9.0	21.0 ± 10.5	0.34	22.8 ± 10.2	19.4 ± 12.3	0.30
HAQ-DI, range 0–3	1.3 [0.5–1.9]	0.4 [0.0–1.8]	0.42	0.8 [0.4–1.4]	1.1 [0.3–1.6]	0.16	1.3 [0.4–1.9]	0.8 [0.3–1.6]	0.29
JAKi: TOF/BAR/PEF/UPA/FIL (%)		21.2/21.2/19.2/15.4/23.1			30.9/22.1/10.3/17.7/19.1-			20.7/25.0/13.0/23.9/17.4-	
csDMARDs: SASP/IGU/BUC/TAC (%)	25.0/29.9/3.2/5.7	32.7/50.0/3.9/9.6	0.17/0.41/0.04/0.15	33.3/46.4/5.8/15.9	38.2/27.9/6.0/11.8	0.10/0.38/0.01/0.12	17.9/38.8/3.0/16.4	28.3/39.1/1.1/10.9	0.25/0.0/0.13/0.16
Glucocorticoid (%), mg/day [IQR]	39.5, 0.0 [0.0–5.0]	42.2, 0.0 [0.0–5.0]	0.06/0.0	34.8, 0.0 [0.0–4.0]	47.0, 1.5 [0.0–5.0]	0.25/0.25	46.2, 0.0 [0.0–2.4]	61.5, 2.0 [0.0–4.5]	0.31/0.51
Steinbrocker stage I/II/III/IV (%)	47.4//21.1/14.0//17.5	47.7/18.2/15.9/18.2	0.06/0.07/0.05/0.02	23.1/24.6/32.3/20.0	55.4/17.9/14.3/12.5	0.67/0.16/0.42/0.20	12.3/21.1/22.8/43.9	24.7/19.2/21.9/34.3	0.31/0.05/9.02/0.20
Steinbrocker class 1/2/3/4 (%)	21.1/41.3/31.2/6.4	23.9/47.8/23.9/4.4	0.07/0.13/0.16/0.09	29.5/54.1/16.4/0.0	11.9/69.5/18.6/0.0	0.43/0.32/0.06/0.0	19.0/55.2/25.9/0.0	18.2/54.6/22.1/5.2	0.02/0.01/0.09/0.33

Values are median [interquartile range] or mean (SD), unless otherwise indicated

*SMD* standardized mean difference, *SAR* Sarilumab, *JAKi* JAK inhibitors, *b/tsDMARDs* biologic/targeted synthetic disease-modifying anti-rheumatic drugs, *RF* rheumatoid factor, *ACPA *anticitrullinated peptide antibody, *CRP* C-reactive protein, *ESR* erythrocyte sedimentation rate, *CDAI* clinical disease activity index, *HAQ-DI* health assessment questionnaire disability index, *TOF* Tofacitinib, *BAR* Baricitinib, *PEF* Peficitinib, *UPA* Upadacitinib, *FIL* Filgotinib, *csDMARDs* conventional synthetic disease-modifying antirheumatic drugs, *SASP* Salazosulfapyridine, *IGU* Iguratimod, *BUC* Bucillamine, *TAC* Tacrolimus

**Table 3:** Baseline clinical data, laboratory data, and SAR and JAKi treatment information of rheumatoid arthritis patients after propensity score matching

	Phase 2 (First-line b/tsDMARDs)			Phase 3 (Second-line b/tsDMARDs)				Phase 3 (≥ Third-line b/tsDMARDs)		
	SAR: *n* = 45	JAKi: *n* = 45	SMD	SAR: *n* = 53	JAKi: *n* = 53	SMD	SAR: *n* = 47		JAKi: *n* = 47	SMD
Age, years	71.2 ± 12.4	71.1 ± 12.7	0.01	67.5 ± 15.8	70.0 ± 10.8	0.19	68.0 ± 13.1		70.6 ± 12.0	0.16
Female (%)	78.0	76.3	0.07	84.9	79.3	0.15	80.9		78.7	0.06
Disease duration, months	34.5 [6.6–169.5]	31.0 [5.0–129.8]	0.04	84.5 [57.6–200.0]	74.0 [35.0–118.5]	0.18	190.0 [121.5–301.0]		168.0 [80.0–216.0]	0.24
RF (%), titer (IU/mL)	71.1, 43.5 [9.3–114.3.3.3]	78.6, 48.1 [14.8–156.9.8.9]	0.17/0.20	83.3, 89.0 [17.2–236.6.2.6]	84.4, 87.0 [19.6–477.0]	0.03/0.30	81.1, 61.0 [26.0–239.0.0.0]		76.3, 122.0 [12.0–661.2.0.2]	0.23/0.02
ACPA (%), titer (IU/mL)	61.7, 17.6 [0.0–286.5.0.5]	64.9, 86.5 [0.4–349.8.4.8]	0.07/0.20	84.4, 111.2 [25.9–342.0]	75.0, 60.4 [3.0–424.5.0.5]	0.01/0.09	76.9 42.1 [3.8–213.2.8.2]		76.2 55.3 [4.0–165.2.0.2]	0.08/0.02
CRP, mg/dL [IQR]	1.1 [0.3–4.0]	1.1 [0.3–4.0]	0.0	0.7 [0.1–1.6]	0.6 [0.0–1.5]	0.09	0.8 [0.2–2.3]		0.8 [0.2–2.2]	0.02
ESR, mm/hr [IQR]	56.0 [23.0–83.0]	39.5 [20.8–70.3]	0.32	39.0 [13.0–67.5]	27.0 [7.0–57.0]	0.31	42.5 [22.3–65.0]		32.0 [12.0–74.0]	0.18
Tender joints, range 0–28 [IQR]	4.0 [1.0–8.0]	3.0 [1.0–7.0]	0.14	4.0 [1.0–6.0]	3.5 [1.0–10.0]	0.21	4.0 [1.0–10.0]		2.0 [1.0–7.0]	0.29
Swollen joints, range 0–28 [IQR]	5.0 [2.0–9.0]	4.0 [1.5–6.0]	0.24	3.0 [1.0–7.0]	4.0 [1.8–6.3]	0.09	5.0 [2.0–7.3]		3.0 [0.0–7.0]	0.31
Patient visual analogue scale, 0–100 mm	61.0 [36.0–78.0]	49.5 [30.0–73.5]	0.23	48.0 [22.0–70.0]	57.5 [33.0–72.0]	0.23	49.0 [25.8–74.5]		63.0 [30.0–78.0]	0.20
Physician visual analogue scale, 0–100 mm	52.0 [34.5–72.0]	43.0 [23.5–66.5]	0.28	43.5 [26.8–53.5]	51.2 [29.3–70.0]	0.35	38.0 [20.0–52.3]		38.0 [25.0–56.0]	0.12
CDAI	22.15 ± 12.7	20.2 ± 8.8	0.16	17.1 ± 8.9	21.1 ± 10.7	0.41	22.3 ± 9.7		20.7 ± 14.5	0.13
HAQ-DI, range 0–3	1.3 [0.4–1.9]	0.2 [0.0–1.8]	0.43	0.7 [0.4–1.3]	1.1 [0.4–1.6]	0.30	1.1 [0.3–1.8]		0.6 [0.1–1.6]	0.27
JAKi: TOF/BAR/PEF/UPA/FIL (%)		22.2/24.4/22.2/15.6/15.6			30.9/22.1/10.3/17.7/19.1-				19.2/27.7/8.5/25.5/19.2	
csDMARDs: SASP/IGU/BUC/TAC (%)	22.2/28.9/4.4/4.4	33.3/46.7/4.4/8.9	0.25.0.37/0.0/0.18	32.1/45.3/9.4/15.1	43.4/28.3/5.7/9.4	0.23/0.35/0.14/0.17	21.3/34.0/4.3/17.0		23.4/38.3/0.0/14.9	0.05/0.09/0.30/0.06
Glucocorticoid (%), mg/day [IQR]	40.0, 0.0 [0.0–3.8]	39.5, 0.0 [0.0–5.0]	0.01/0.12	35.9, 0.0 [0.0–4.0]	50.0, 0.0 [0.0–5.0]	0.29/0.09	44.7, 0.0 [0.0–2.0]		51.1, 0.0 [0.0–3.3]	0.13/0.21
Steinbrocker stage I/II/III/IV (%)	36.6/19.5/17.1/26.8	51.2/17.1/14.6/17.1	0.29/0.06/0.07/0.23	10.3/27.6/27.6/34.5	33.3/23.8.21.4/21.4	0.56/0.09/0.14/0.29	14.6/19.5/26.8/39.0		22.5/17.5/25.0/35.0	0.20/0.05/0.04/0.08
Steinbrocker class 1/2/3/4 (%)	24.4/41.5/29.3/4.9	25.0/47.5/22.5/5.0	0.01/0.12/0.16/0.01	29.2/56.3/14.6/0.0	10.6/68.1/21.3/0.0	0.47/0.24/0.18/0.0	19.5/56.1/24.4/0.0		18.0/59.0/20.5/2.6	0.04/0.06/0.09/0.23

Values are median [interquartile range] or mean (SD), unless otherwise indicated

*SMD* standardized mean difference, *SAR* Sarilumab, *JAKi* JAK inhibitors, *b/tsDMARDs* biologic/targeted synthetic disease-modifying anti-rheumatic drugs, *RF* rheumatoid factor, *ACPA* anticitrullinated peptide antibody, *CRP* C-reactive protein, *ESR* erythrocyte sedimentation rate, *CDAI* clinical disease activity index, *HAQ-DI* health assessment questionnaire disability index, *TOF* Tofacitinib, *BAR* Baricitinib, *PEF* Peficitinib, *UPA* Upadacitinib, *FIL* Filgotinib, *csDMARDs* conventional synthetic disease-modifying antirheumatic drugs, *SASP* Salazosulfapyridine, *IGU* Iguratimod, *BUC* Bucillamine, *TAC* Tacrolimus

### Overall clinical outcomes

**Fig. 1:**
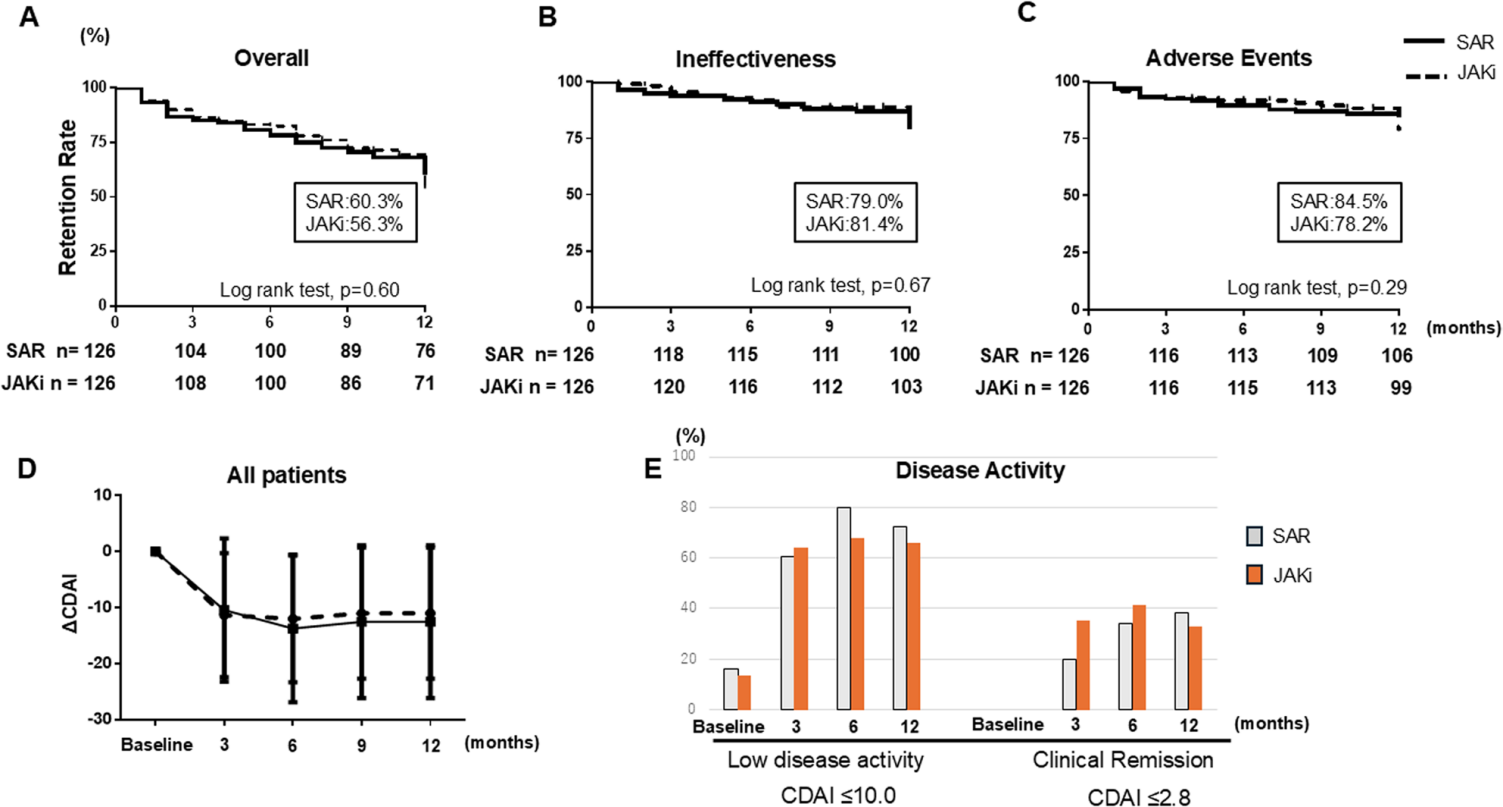
Overall clinical outcomes over 12 months in patients treated with SAR or JAKi. Kaplan–Meier curves showing treatment overall (**A**), ineffectiveness (**B**), and adverse events (**C**) retention rates. CDAI values at baseline, 3, 6, 9, and 12 months (**D**). Proportion of patients achieving CDAI LDA (LDA, CDAI ≤ 10.0) and clinical remission (CDAI ≤ 2.8) (**E**). Abbreviations: SAR, sarilumab; JAKi, Janus kinase inhibitors; CDAI, Clinical Disease Activity Index; LDA, low disease activity

Following PSM, we first compared the primary outcome of 12-month drug retention between the SAR and JAKi groups and then evaluated secondary clinical outcomes over 12 months. Figure 1A shows the overall retention rates, including all reasons for treatment discontinuation. At 6 months, the retention rates were 82.5% for the JAKi group and 78.3% for the SAR group. At 12 months, the rates declined to 56.3% and 60.3% for the JAKi and SAR groups, respectively. Kaplan–Meier analysis revealed no statistically significant difference in treatment continuation between the groups (log-rank test, *p* = 0.60). In a Cox proportional hazards model, the point estimates slightly favored SAR, but the difference was not statistically significant (hazard ratio [HR] 1.1, 95% confidence interval [CI] 0.75–1.66, *p* = 0.60). In Fig. 1B, time to discontinuation for ineffectiveness did not differ between SAR and JAKi (log-rank *p* = 0.67), with survival curves remaining closely overlapping throughout follow-up. For discontinuation due to AEs, no statistically significant difference was observed between the two groups (log-rank *p* = 0.29). As illustrated in Fig. 1D, both groups demonstrated substantial improvements in CDAI from baseline at 3, 6, and 12 months. The differences in between-group in mean CDAI at each time point were small and not statistically significant (all *p* > 0.05), indicating that a clear advantage of either treatment in terms of disease activity could not be demonstrated. Consistently, rates of CDAI low disease activity (LDA; CDAI ≤ 10.0) and remission (CDAI ≤ 2.8) did not differ significantly between groups at 3, 6, and 12 months (Fig. 1E). At 12 months, the proportions achieving LDA were 72.3% in the SAR group and 65.9% in the JAKi group, and the proportions achieving remission were 38.3% and 33.0%, respectively, none of these differences reached statistical significance.

### Phase-based clinical outcomes in MTX-free RA with sarilumab versus JAK inhibitors by prior b/tsDMARD use

**Fig. 2:**
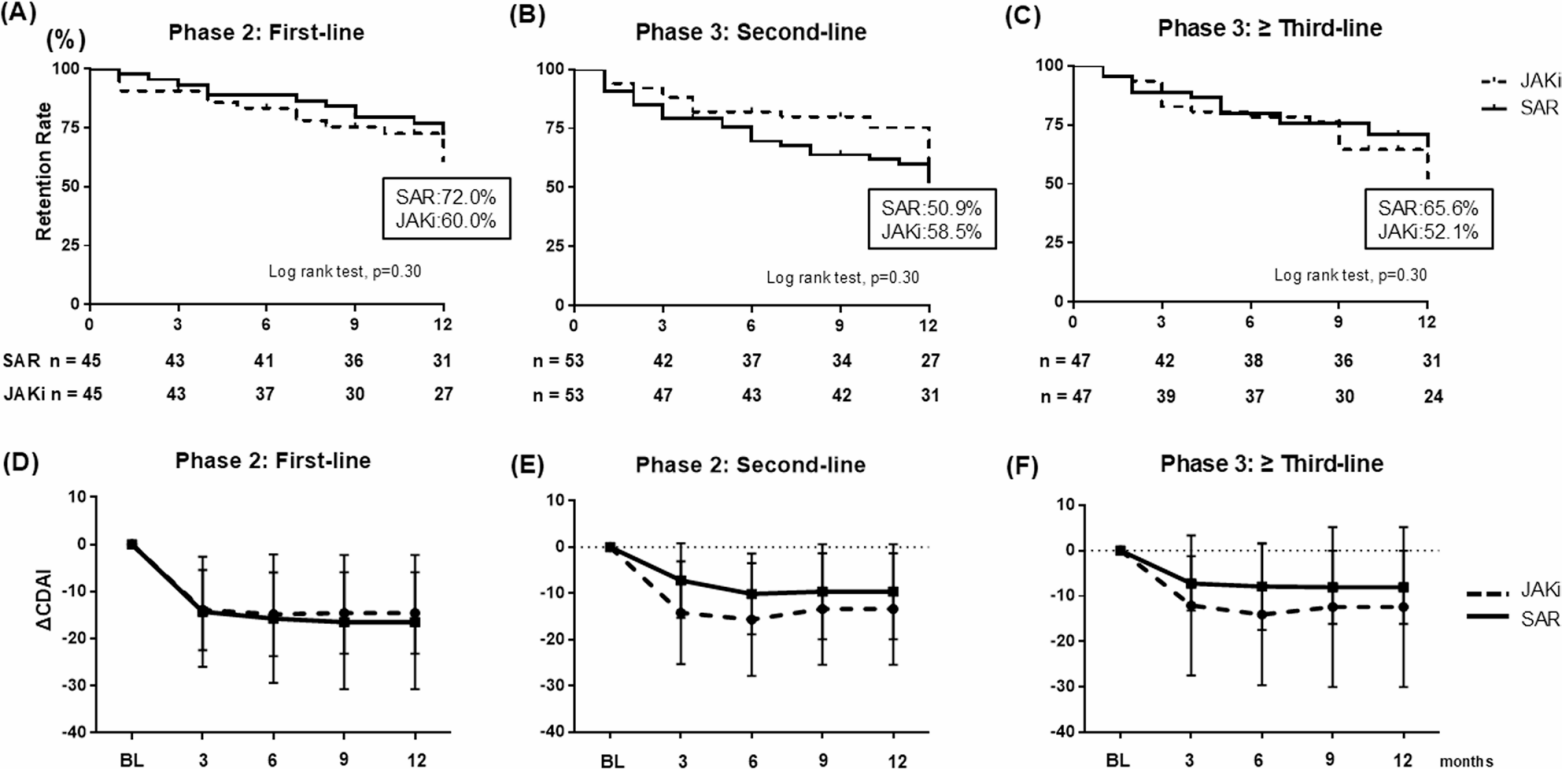
Phase-based treatment outcomes in methotrexate-free RA stratified by prior b/tsDMARD use. **A**–**C** Kaplan–Meier curves showing treatment retention rates in Phase 2 first-line, Phase 3 s-line, and third-line or later settings. **D**–**F** Changes in CDAI from baseline to 12 months. Abbreviations:RA, rheumatoid arthritis; BL, baseline; SAR, sarilumab; JAKi, Janus kinase inhibitors; b/tsDMARDs, biologic/targeted synthetic disease-modifying antirheumatic drugs; CDAI, Clinical Disease Activity Index

We evaluated 12-month drug retention and ΔCDAI according to prior treatment line (Fig. 2). Across first-, second-, and ≥ third-line strata, retention did not differ significantly between SAR and JAKi (all log-rank *p* = 0.30). In first-line therapy (Phase 2; Fig. 2A and D), 12-month retention was 72.0% with SAR and 60.0% with JAKi, with similar ΔCDAI at 6 and 12 months (− 14.3 vs. − 13.9 and − 16.5 vs. − 14.5, respectively). In second-line therapy (Phase 3; Fig. 2B and E), 12-month retention was 50.9% vs. 58.5%, with numerically larger ΔCDAI for JAKi (− 10.1 vs. − 15.6 at 6 months and − 9.6 vs. − 13.4 at 12 months). In ≥ third-line therapy (Phase 3; Fig. 2C and F), 12-month retention was 65.6% vs. 52.1%, while ΔCDAI again tended to be numerically greater with JAKi (− 7.9 vs. − 14.1 at 6 months and − 8.1 vs. − 12.4 at 12 months). Taken together, these phase-based analyses show that both SAR and JAKi achieved clinically meaningful improvements in CDAI across prior-line strata, while between-group differences in retention and ΔCDAI were modest, inconsistent in direction, and not statistically significant.

### Glucocorticoid outcomes over 12 months in sarilumab and JAK inhibitors by phase and treatment-line

**Fig. 3:**
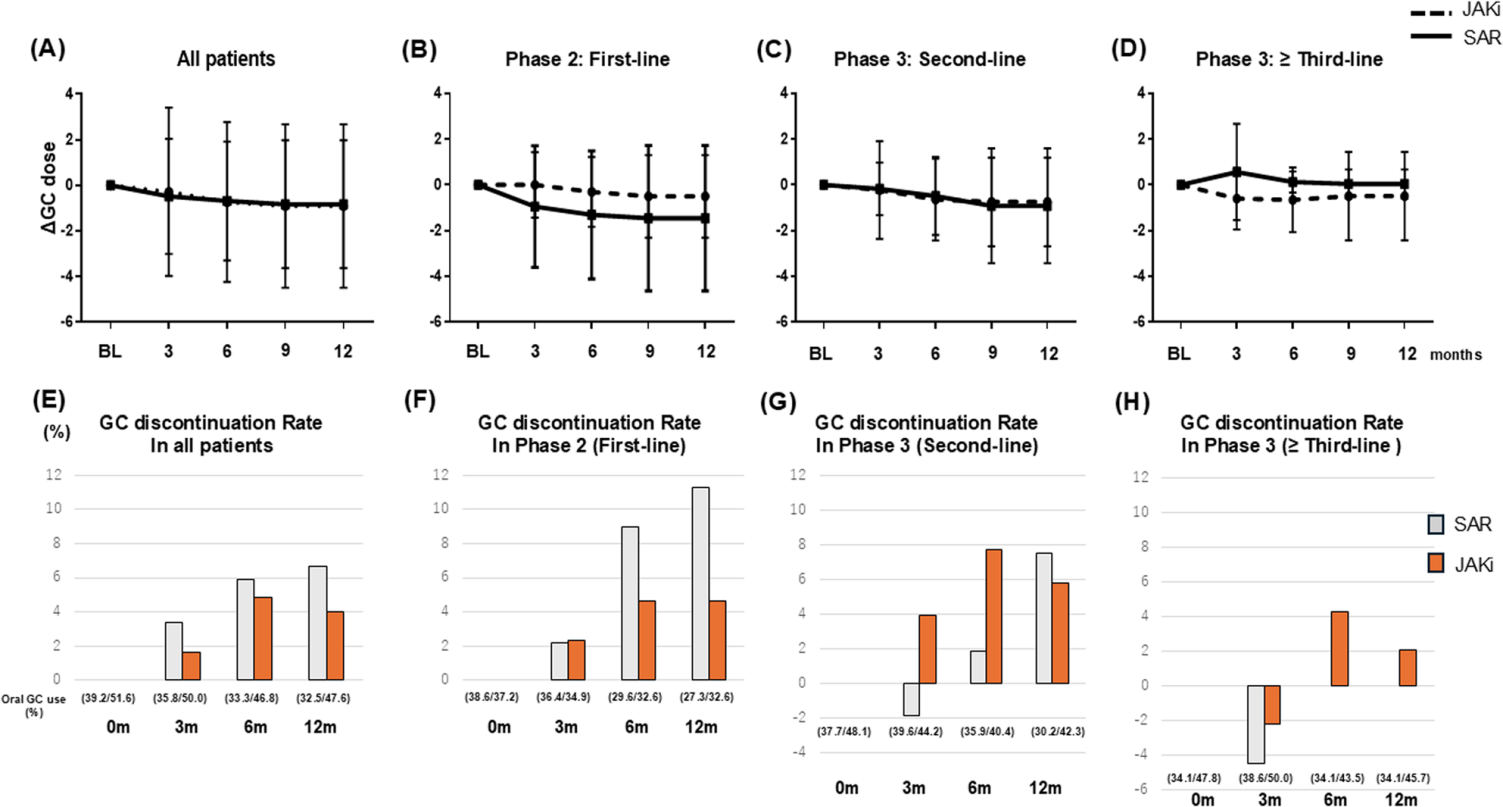
Phase-based changes in glucocorticoid (GC) dose (ΔGC dose) in methotrexate-free RA stratified by prior b/tsDMARD use. **A**–**D** Median changes in glucocorticoid (GC) dose (ΔGC dose) over 12 months, stratified by prior b/tsDMARD use. **E**–**H** Discontinuation rate of oral GC over the observation period from baseline to 12 months in Phase 2 first-line, Phase 3 s-line, and third-line or later settings. GC outcomes were analyzed on an observed-case basis, patients contributed data up to treatment discontinuation, and no imputation was performed thereafter. All patients in the PSM cohort were included in the main analyses. Patients not receiving GC at baseline were assigned a daily dose of 0 mg/day and were considered already off GC for the calculation of discontinuation proportions. Because GC dose distributions were highly skewed with many zero values, changes are presented as medians with interquartile ranges. Abbreviations: RA, rheumatoid arthritis; GC, glucocorticoids; SAR, sarilumab; JAKi, Janus kinase inhibitors, b/tsDMARDs, biologic/targeted synthetic disease-modifying antirheumatic drugs

Figure 3 summarizes oral GC outcomes from baseline to 12 months. Figure 3A–D show changes in daily GC dose, and Fig. 3E–H show cumulative GC discontinuation. In the overall cohort (Fig. 3A and E), median daily GC dose remained low and largely unchanged (0.0 [0.0–0.0] mg/day in both groups at 3 months; 0.0 [0.0–0.0] with SAR and 0.0 [− 1.0–0.0] with JAKi at 12 months), while the proportion of patients off GC increased from 3.3% to 6.7% with SAR and from 1.6% to 4.0% with JAKi. In first-line therapy (Phase 2; Fig. 3B and F), 12-month median dose changes were 0.0 [− 2.0–0.0] vs. 0.0 [0.0–0.0] for SAR and JAKi, respectively, and GC discontinuation rose from 2.2% to 11.3% with SAR and from 2.3% to 4.6% with JAKi. In second-line (Phase 3; Fig. 3C and G) and ≥ third-line therapy (Phase 3; Fig. 3D and H), median doses remained 0.0 [0.0–0.0] in both groups and GC discontinuation increased modestly (to 7.5% vs. 5.8% and 0.0% vs. 2.1% for SAR vs. JAKi, respectively). Overall, these data indicate stable to modestly decreasing daily GC doses with gradual increases in discontinuation, without a clear or consistent divergence between SAR and JAKi within prior-line strata. GC outcomes were evaluated on an observed-case basis without imputation after treatment discontinuation, and the main analyses included all patients in the matched cohort, regardless of GC use at baseline, to reflect real-world prescribing patterns.

### CDAI improvement stratified by baseline prognostic factors

**Fig. 4:**
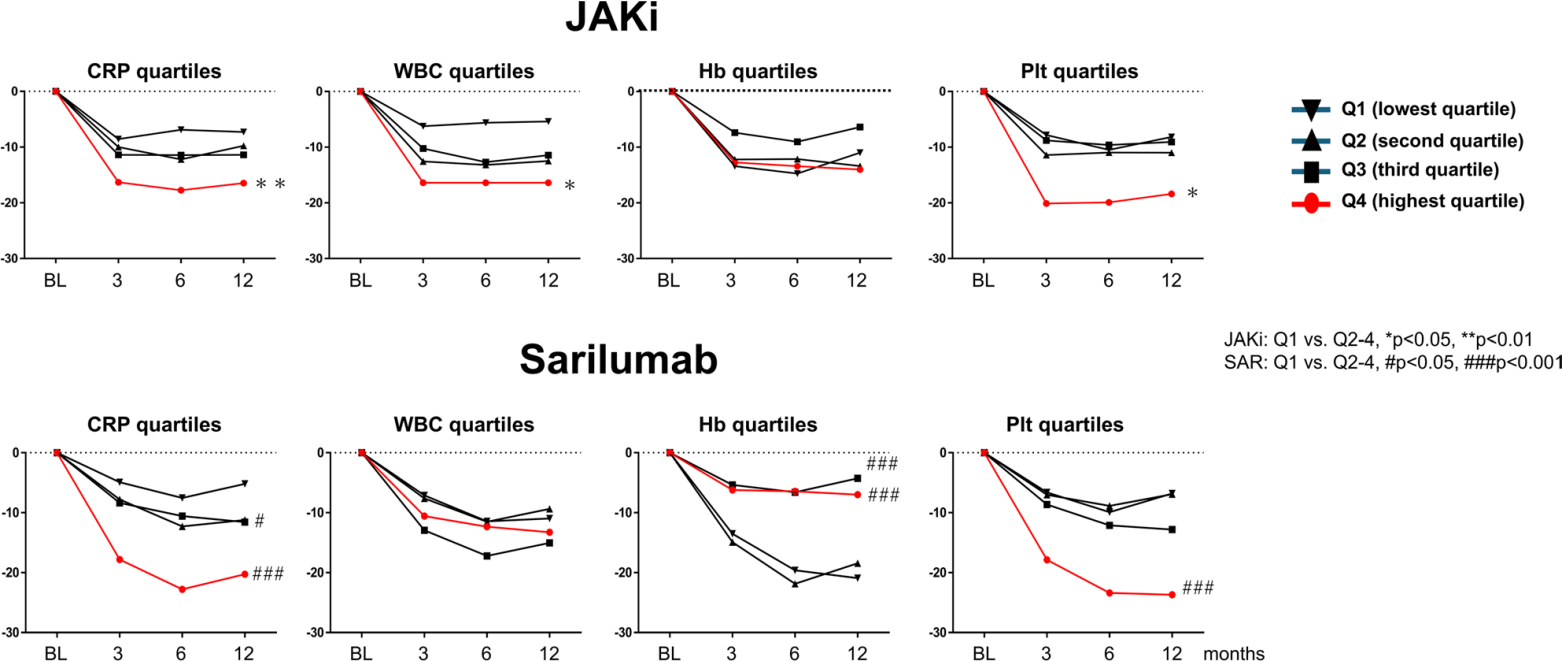
CDAI changes (ΔCDAI) from baseline to 12 months, stratified by quartiles of prognostic factors. Median CDAI reductions by quartile of baseline CRP, WBC, Hb, and Plt in JAKi and SAR. Abbreviations: SAR, sarilumab; JAKi, Janus kinase inhibitors; CDAI, Clinical Disease Activity Index; CRP, C-reactive protein; WBC, white blood cell count; Hb, hemoglobin; Plt, Plt count; Q1–Q4, quartile 1 to quartile 4

**Fig. 5:**
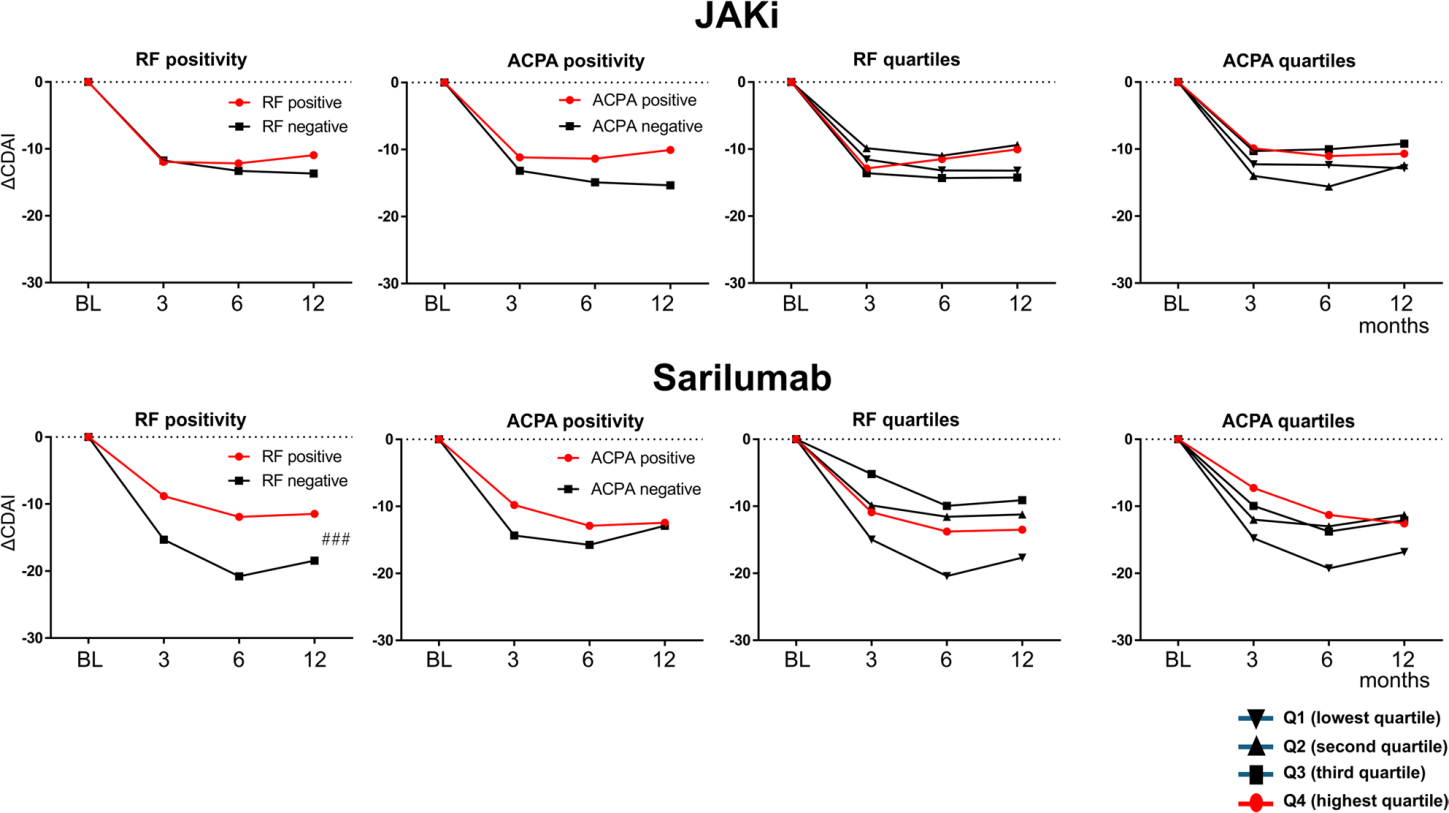
CDAI changes (ΔCDAI) from baseline to 12 months, according to RF and ACPA positivity and titers. Median CDAI reductions by quartile of baseline RF and ACPA positivity, RF and ACPA titers in JAKi and SAR. Abbreviations: SAR, sarilumab; JAKi, Janus kinase inhibitors; CDAI, Clinical Disease Activity Index; RF, rheumatoid factor; ACPA, anti-citrullinated peptide antibody; Q1–Q4, quartile 1 to quartile 4

ΔCDAI at 12 months was evaluated after stratifying baseline CRP, RF, ACPA, WBC, Hb, and Plt into quartiles (Suppl. Tables 1 and Fig. 4). For CRP, improvement rose across quartiles in both groups. In SAR, changes were − 5.2, − 11.2, − 11.6, and − 20.3 from Q1 to Q4; in JAKi, − 7.2, − 9.8, − 11.4, and − 16.5, with Q4 greater than Q1 (*p* < 0.05) in JAKi. For WBC, only JAKi showed a quartile effect (Q4 − 17.5 vs. Q1 − 5.4; *p* < 0.05). For Hb, SAR showed greater improvement in Q1 (7.3–<10.5 g/dL) than in Q3 and Q4 (− 20.9 vs. −4.3 and − 7.0; *p* < 0.001), whereas no Hb-related differences were seen with JAKi. For Plt, both groups improved more in Q4 than Q1 (SAR − 24.0 vs. −6.7; JAKi − 18.4 vs. −8.2; both *p* < 0.05). By serological status (Fig. 5), RF-negative patients on SAR improved more than RF-positive patients (− 18.5 vs. −11.5; *p* < 0.001). ACPA status did not materially affect ΔCDAI in either group. Quartile analyses of RF and ACPA titers showed no statistically significant differences, although numerically larger improvements were seen in the lowest quartiles.

### Factors associated with achieving low disease activity at 12 months

Baseline predictors of achieving CDAI-LDA (CDAI ≤ 10) at 12 months were evaluated by logistic regression conducted separately for SAR and JAKi in the matched cohort (Table 4). In the SAR group, younger age (adjusted odds ratio [OR] 0.98; 95% CI 0.96–0.99; *p* = 0.01), b/tsDMARD-naïve status (adjusted OR 2.59; 95% CI 2.21–3.70; *p* = 0.01), lower Hb (adjusted OR 0.54; 95% CI 0.31–0.95; *p* = 0.05), higher Plt (adjusted OR 1.24; 95% CI 1.12–1.37; *p* = 0.001), and higher CRP (adjusted OR 1.83; 95% CI 1.32–2.45; *p* = 0.001) were independently associated with LDA. In the JAKi group, higher Plt (adjusted OR 1.16; 95% CI 1.07–1.28; *p* = 0.001) and higher CRP (adjusted OR 1.23; 95% CI 1.16–1.78; *p* = 0.03) remained independent predictors.

**Table 4:** Baseline predictors of achieving CDAI low disease activity at 12 months within the propensity score–matched cohort

	SAR				JAKi			
	univariable analysis		multivariable analysis		univariable analysis		multivariable analysis	
	Odds Ratio (95% CI)	*p*-value	Odds Ratio (95% CI)	*p*-value	Odds Ratio (95% CI)	*p-*value	Odds Ratio (95% CI)	*p*-value
Age, years	0.97 (0.96–0.99)	0.001	0.98 (0.96–0.99)	0.01	0.82 (0.65–1.24)	0.82		
Female	0.84 (0.66–2.25)	0.57			0.75 (0.38–1.40)	0.37		
Disease duration, months	1.0 (0.99–1.00.99.00)	0.13			0.92 (0.50–1.70)	0.77		
b/tsDMARDs naive	2.46 (1.46–4.33)	0.001	2.59 (2.21–3.70)	0.01	2.63 (1.56–4.6)	0.001	1.5 (0.75–2.66)	0.26
RF positivity	0.69 (0.28–0.92)	0.02	0.34 (0.69–1.61)	0.39	1.58 (0.99–2.48)	0.05	1.43 (0.24–8.52)	0.70
ACPA positivity	0.95 (0.25–2.05)	0.003	0.64 (0.83–2.21)	0.16	1.74 (1.09–2.77)	0.02	1.19 (0.29–5.00.29.00)	0.58
WBC	1.05 (0.70–1.56)	0.82			0.59 (0.39–0.89)	0.001	1.00 (0.99–1.00.99.00)	0.20
Hb	0.37 (0.22–0.60)	0.001	0.54 (0.31–0.95)	0.05	0.24 (0.14–0.41)	0.001	1.50 (0.98–2.25)	0.07
Plt	1.94 (1.54–2.70)	0.001	1.24 (1.12–1.37)	0.001	1.53 (1.30–1.93)	0.003	1.16 (1.07–1.28)	0.001
CRP	2.25 (1.81–2.98)	0.001	1.83 (1.32–2.45)	0.001	1.45 (1.12–1.87)	0.02	1.23 (1.16–1.78)	0.03
Concomitant of GC (%)	0.71 (0.47–1.08)	0.11			0.67 (0.44–1.02)	0.06		

Analyses were performed within the propensity score–matched cohort (*n* = 252; 126 SAR and 126 JAKi). Analysis of factors associated with achieving low disease activity in SAR and JAKi. Values are median [25th–75th centiles] or mean (SD), unless otherwise indicated

*SAR* Sarilumab, *JAKi* Janus kinase inhibitors, *bDMARDs* biological disease-modifying antirheumatic drugs, *tsDMARDs* targeted synthetic disease-modifying antirheumatic drugs, *RF* rheumatoid factor, *ACPA* anticitrullinated peptide antibody, *WBC* white blood cell, *Hb* Hemoglobin, *Plt* Platelet, *CRP* C-reactive protein, *GC* glucocorticoid,

*p < 0.05, **p < 0.01, ***p < 0.001

In univariate analyses, additional factors were associated with LDA (including RF positivity, b/tsDMARD-naïve status, and lower WBC and Hb), but these did not remain significant after multivariable adjustment. Overall, these results indicate that markers of systemic inflammation and hematologic activity—particularly elevated Plt and CRP—are consistently linked to better treatment response in MTX-free regimens, whereas other baseline characteristics lose significance once confounding factors are accounted for.

### Safety outcomes and adverse events leading to treatment discontinuation

Table 5 presents safety outcomes in the matched cohorts (*n* = 126 per group). Any AE occurred in 22 of 126 patients (17.5%, 95% CI 10.9–24.1) with SAR and in 42 of 126 (33.3%, 95% CI 25.1–41.5) with JAKi, an absolute difference of 15.8% points, corresponding to an approximately 1.9-fold higher risk with JAKi. Infections leading to discontinuation were observed in 3.2% (95% CI 0.1–6.3) of SAR-treated patients and 10.3% (95% CI 5.0–15.6) of JAKi-treated patients, and herpes zoster in 0.0% (95% CI 0.0–2.4) vs. 2.4% (95% CI 0.0–5.1), respectively. Other specific AEs leading to discontinuation, including cancer, rash, RA-ILD exacerbation, renal injury, bone marrow suppression, and miscellaneous events, were rare in both groups (all ≤ 5%) with wide CIs and detailed proportions and 95% CIs are provided.

**Table 5:** Safety and adverse events leading to discontinuation among RA patients on sarilumab and JAK inhibitors

	Sarilumab		JAK inhibitors	
	Events (%)	95% confidence interval (95% CI)	Events (%)	95% confidence interval (95% CI)
All patients	126		126	
Any adverse events	22 (17.5)	10.9–24.1	42 (33.3)	25.1–41.5
Infection	4 (3.2)	0.1–6.3	13 (10.3)	5.0–15.6.0.6
Herpes zoster	0 (0.0)	0.0–2.4.0.4	3 (2.4)	0.0–5.1.0.1
Cancer	1 (0.8)	0.3–2.8	2 (1.6)	0.0–3.8.0.8
Rash	3 (2.4)	0.0–5.1.0.1	1 (0.8)	0.3–2.4
Exacerbation of RA-ILD	1 (0.8)	0.2–1.4	2 (1.6)	1.0–3.8.0.8
Renal injury	0, 0.0	0.0–2.4.0.4	2 (1.6)	1.0–3.1.0.1
Bone marrow suppression	4 (3.2)	0.1–6.3	3 (2.4)	2.0–4.8.0.8
Others	6 (4.8)	1.1–8.5	0 (0.0)	0.0–2.4.0.4

*JAK* Janus kinase, *RA-ILD* rheumatoid arthritis–associated interstitial lung disease

## Discussion

This multicenter real-world study compared SAR, an IL-6Ri, and JAKi in MTX-free RA and did not demonstrate a clear difference in overall clinical effectiveness, while showing variability in response according to patient phenotype. Across the cohort, CDAI improvement was broadly comparable between SAR and JAKi, while baseline hematologic and serologic features modified the magnitude of response. These observations support a precision medicine approach in MTX-free care in which routinely available laboratory measures inform initial treatment selection. Patients with low baseline hemoglobin experienced greater improvement with SAR, which is consistent with the biology of IL-6–driven anemia. IL-6 upregulates hepatic hepcidin, disrupts iron handling, and contributes to anemia of chronic disease, and IL-6 receptor inhibition can mitigate this process [18]. The larger CDAI improvement observed in the lowest hemoglobin quartile aligns with preferential effectiveness of IL-6Ri in an IL-6–dominant hematologic phenotype and is concordant with prior reports that hemoglobin recovery during IL-6Ri treatment tracks with disease control [19].

Plt was associated with greater clinical improvement across both SAR and JAKi. In univariate and multivariable models, higher baseline Plt predicted better response within each class. Any apparent advantage for SAR was modest with overlapping confidence intervals and should be considered exploratory. This pattern is biologically plausible because IL-6 promotes thrombopoiesis and thrombocytosis mirrors inflammatory activity in RA [20], and Plt often decline after IL-6 receptor inhibition in parallel with clinical improvement [21]. Platelet-derived mediators such as sCD40L can amplify synovial inflammation through fibroblast-like synoviocyte activation and induction of IL-6, establishing a feed-forward loop [22, 23]. Reductions in circulating sCD40L after IL-6Ri have been reported and correlate with improvement in disease activity [24]. Taken together, elevated Plt should be viewed not as a selective indicator for SAR but as a hematologic marker of active inflammation that may forecast response to either class while potentially enriching for IL-6 biology in a subset.

CRP emerged as an independent predictor of achieving CDAI low disease activity in both treatment groups, consistent with CRP as an integrative measure of systemic inflammation responsive to either pathway [25–27]. Based on this finding, patients with high CRP and clinically high disease activity by CDAI should be considered priority treatment targets in MTX-free settings for both SAR and JAKi. When this inflammatory state coexists with low Hb or high Plt that suggest an IL-6–dominant hematologic phenotype SAR may provide greater benefit. In highly inflammatory cases without these hematologic features JAKi remain a reasonable option. For either class treatment intensity should be optimized on the basis of baseline CRP and CDAI, and structured tapering and discontinuation of oral glucocorticoids is recommended.

Prior studies have suggested that IL-6 receptor inhibition is effective in both seropositive and seronegative RA, and that some patients with seronegative RA may exhibit particularly IL-6–driven disease biology. Tocilizumab has shown comparable, and in some cohorts even numerically greater, clinical improvement in seronegative RA compared with seropositive RA, and observational data indicate that IL-6R therapies maintain good effectiveness regardless of serological status. Serological status may also influence the magnitude of treatment response, particularly for IL-6Ri. In our cohort, RF-negative patients treated with SAR had baseline CDAI levels similar to those of RF-positive patients but exhibited numerically larger absolute reductions in CDAI over 12 months. This pattern aligns with prior reports that IL-6 receptor inhibitors are effective in both seropositive and seronegative RA, including data showing that tocilizumab improves disease activity irrespective of serologic status for RF and/or ACPA [28], and may be particularly beneficial in subsets of seronegative disease with IL-6–driven biology. At the same time, the number of patients contributing to these subgroup analyses was limited, and the interaction between RF status and treatment response was examined only exploratorily; thus, this finding should be regarded as hypothesis-generating rather than definitive evidence of effect modification. We did not assess detailed mechanistic biomarkers such as IL-6/STAT signaling in this study, and unmeasured factors associated with serological status (including comorbidity patterns, pain perception, or health-related quality of life) may also have contributed to the observed differences in CDAI trajectories. Further biomarker-stratified studies are therefore required to clarify whether RF or ACPA status truly modifies the response to IL-6Ri.

Phase-based analyses also provided clinical context. In first-line therapy, retention rates were numerically higher with SAR, whereas CDAI improvement was similar between the two groups. Differences were small in second-line and in third-line or later. These patterns suggest that prior treatment exposure and baseline inflammatory biology may be more informative than drug class alone when MTX is not used. Glucocorticoid outcomes were concordant with these findings. Daily oral doses were stable to modestly decrease over twelve months and the proportion discontinuing glucocorticoids rose gradually in both groups. In first-line use SAR was associated with greater glucocorticoid discontinuation which may have contributed to better persistence. These observations are hypothesis generating and require confirmation.

Safety findings were directionally different yet statistically underpowered. After matching, discontinuations due to infection occurred in 3.2% with SAR versus 10.3% with JAKi and herpes zoster in 0.0% versus 2.4% respectively. Event counts were small and confidence intervals were wide, so the study was not powered for definitive safety comparisons. Even so, the data support routine infection risk assessment at initiation and during follow-up, review of vaccination status for herpes zoster where available, careful monitoring in older adults, and attention to background glucocorticoid exposure.

This study has limitations. The design was retrospective and observational, so residual confounding and treatment selection bias may persist despite 1:1 propensity score matching and multivariable adjustment. Some covariates remained imbalanced after matching and drug-specific strata within the JAKi class were small, limiting the robustness of subgroup inferences. The IL-6Ri group comprised only sarilumab, and data were derived from a limited number of participating centers, which may restrict generalizability to other IL-6Ri agents and broader practice settings. Predictor analyses were performed within the matched cohort with additional multivariable adjustment and should therefore be interpreted as adjusted associations within this population rather than as independent confirmation in a separate dataset; residual confounding cannot be fully excluded. In exploratory analyses using the crude (unmatched) cohort, the directions and approximate magnitudes of associations were broadly similar but less stable (data not shown), so we present the matched-cohort results as our primary analyses. In addition, some eligible patients were excluded from the matched analyses because of missing baseline covariates. Of 472 patients who initiated SAR or JAKi, 40 (8.5%) were excluded owing to missing key variables. Baseline characteristics were broadly similar between included patients and those excluded for missing covariates (Suppl. Table 2), suggesting that missingness was approximately random, although selection bias related to missing data cannot be ruled out. Another limitation is that we performed numerous stratified and quartile-based subgroup analyses without formal adjustment for multiple comparisons; these analyses were prespecified but exploratory, and the corresponding findings should be regarded as hypothesis-generating and interpreted with caution. Safety analyses were also limited by small event counts and wide CIs, such that the study was not powered for formal between-group safety comparisons. Several AEs, including herpes zoster and renal injury in the SAR group, had very low event counts, with some categories showing zero occurrences, although 95% CIs were provided for these zero-event cells (e.g. 0.0% with an upper 95% CI of 2.4%), the resulting wide intervals constrain the precision of between-group comparisons for uncommon safety outcomes, and larger cohorts or pooled analyses will be required to more accurately assess rare AEs. Finally, CDAI trajectories were analyzed on an observed-case basis without imputation, because treatment discontinuation may be related to lack of efficacy or AEs, informative missingness cannot be excluded, and the true magnitude of CDAI changes among patients who discontinued treatment may differ from the observed estimates. These results have practical implications. Baseline WBC, Hb, Plt, CRP, and serologies including RF and ACPA can be incorporated into routine triage for MTX-free regimens. In patients with inflammatory anemia or thrombocytosis both SAR and JAKi are reasonable options. When thrombocytosis coexists with low Hb or other features suggestive of IL-6-dominant biology SAR may be prioritized, whereas in thrombocytosis without such features either class can be selected through shared decision-making that considers comorbidity profile and treatment access. Regardless of class, structured efforts to taper and discontinue GCs appear feasible and may support treatment persistence.

Future research should include prospective biomarker-stratified trials that compare SAR and JAKi in MTX-free RA with a focus on patients with anemia, thrombocytosis, or high CRP. Studies should incorporate IL-6–related biomarkers including hepcidin, ferritin, and transferrin saturation and should evaluate radiographic and functional outcomes along with safety signals with adequate power. Validation of platelet-derived mediators as predictive or pharmacodynamic biomarkers and formal testing of treatment by biomarker interactions are also warranted.

In summary, SAR and JAKi achieved broadly similar clinical effectiveness in MTX-free RA. Signals suggest that IL-6 receptor inhibition may confer greater benefit in patients with low Hb and possibly in subsets with IL-6-enriched biology while both classes appear effective in patients with thrombocytosis. First-line use of SAR was associated with glucocorticoid sparing. These findings support a pragmatic precision approach that uses routine laboratory data including CRP and CDAI to guide class selection while highlighting the need for prospective confirmation.

## Supplementary Information

Supplementary Material 1.

Supplementary Material 2.

## Data Availability

The datasets used and analyzed in this study are available from the corresponding author upon reasonable request.

## Abbreviations

RA: Rheumatoid arthritis
MTX: Methotrexate
SAR: Sarilumab
JAKi: Janus kinase inhibitor
DMARDs: Disease modifying antirheumatic drugs
csDMARDs: conventional synthetic DMARDs
bDMARDs: biologic DMARDs
tsDMARDs: targeted synthetic DMARDs
b/tsDMARDs: biologic or targeted synthetic DMARDs
CDAI: Clinical Disease Activity Index
LDA: Low disease activity (CDAI ≤10)
GC: Glucocorticoid
AE(s): Adverse event(s)
RA-ILD: Rheumatoid arthritis associated interstitial lung disease
IL-6: Interleukin 6
IL-6R: Interleukin 6 receptor
IL-6Ri: Interleukin 6 receptor inhibitor
CRP: C reactive protein
ESR: Erythrocyte sedimentationrate
Hb: Hemoglobin
WBC: White blood cell
Plt: Platelet counts
VAS: Visual analogue scale
HAQ-DI: Health Assessment Questionnaire Disability Index
PSM: Propensity score matching
SMD: Standardized mean difference
KM: Kaplan–Meier
HR: Hazard ratio
OR: Odds ratio
CI: Confidence interval
SD: Standard deviation
IQR: Interquartile range
Q1–Q4: Quartiles 1 through 4
TOF: Tofacitinib
BAR: Baricitinib
PEF: Peficitinib
UPA: Upadacitinib
FIL: Filgotinib
