# Supplementary material for: Real-world comparative effectiveness of sarilumab versus Janus kinase inhibitors as monotherapy in rheumatoid arthritis
Source: Arthritis Res Ther. 2026 Jan 2;28:32. doi: 10.1186/s13075-025-03722-5 (PMC12888330; doi:10.1186/s13075-025-03722-5)
Supplement: Supplementary file 1 — Supplementary Material 1. [file 13075_2025_3722_MOESM1_ESM.docx]

**Supplementary Table 1.** Patient background after matching in rheumatoid arthritis patients with SAR and JAKi group without methotrexate treatment.

|  | **Q1** | **Number** | **Q2** | **Number** | **Q3** | **Number** | **Q4** | **Number** |
| --- | --- | --- | --- | --- | --- | --- | --- | --- |
| SAR |  |  |  |  |  |  |  |  |
| CDAI (n=117) | ≤12.4 | 30 | 12.5–<18.1 | 29 | 18.1–<27.0 | 29 | ≥27.0 | 29 |
| CRP (n=124) | ≤0.228 | 31 | 0.229–<1.456 | 31 | 1.456–<4.247 | 31 | ≥4.247 | 31 |
| WBC (n=99) | 2280–<5700 | 27 | 5700–<7500 | 26 | 7500–<9500 | 26 | 9500–≤14950 | 26 |
| Hb (n=83) | 7.3–<10.5 | 20 | 10.5–<11.9 | 21 | 11.9–<12.9 | 19 | 12.9–<15.6 | 23 |
| Plt (n=105) | ≤20.2 | 28 | 20.3–<25.9 | 25 | 25.9–<33.2 | 27 | ≥33.2 | 25 |
| RF (n=99) | ≤19.0 | 26 | 19.1–<59.1 | 24 | 59.1–<172.1 | 24 | ≥172.1 | 25 |
| ACPA (n=117) | ≤0.6 | 30 | 0.7–<48.1 | 29 | 48.1–<230.0 | 29 | ≥230.0 | 29 |
|  |  |  |  |  |  |  |  |  |
| JAKi |  |  |  |  |  |  |  |  |
| CDAI (n=102) | 3.0–<13.35 | 26 | 13.35–<18.0 | 26 | 18.0–<24.8 | 25 | 24.8–48.3 | 25 |
| CRP (n=122) | ≤0.0562 | 31 | 0.0563–<0.6641 | 30 | 0.6641–<1.950 | 30 | ≥1.950 | 31 |
| WBC (n=121) | 1790–<5380 | 30 | 5380–<7430 | 30 | 7430–<9300 | 30 | 9300–≤16060 | 31 |
| Hb (n=110) | 7.7–<10.3 | 30 | 10.3–<11.9 | 27 | 11.9–<13.2 | 25 | 13.2–<17.6 | 28 |
| Plt (n=120) | 9.4–<19.1 | 30 | 19.1–<23.9 | 30 | 23.9–<30.0 | 30 | 30.0–46.2 | 30 |
| RF (n=103) | ≤19.0 | 26 | 19.1–<59.1 | 24 | 59.1–<172.1 | 24 | ≥172.1 | 25 |
| ACPA (n=94) | ≤30.8 | 24 | 30.9–<87.4 | 24 | 87.4–<315.0 | 22 | ≥315.0 | 24 |

SAR: Sarilumab; JAKi: Janus kinase inhibitors; CDAI: Clinical Disease Activity Index; CRP: C-reactive protein, WBC: white blood cell count; Net: neutrophil count; Hb: hemoglobin; Plt: platelet count, RF: rheumatoid factor; ACPA: anti-citrullinated peptide antibody
