# Supplementary material for: Real-world comparative effectiveness of sarilumab versus Janus kinase inhibitors as monotherapy in rheumatoid arthritis
Source: Arthritis Res Ther. 2026 Jan 2;28:32. doi: 10.1186/s13075-025-03722-5 (PMC12888330; doi:10.1186/s13075-025-03722-5)
Supplement: Supplementary file 2 — Supplementary Material 2. [file 13075_2025_3722_MOESM2_ESM.docx]

**Supplementary Table 2.** Baseline characteristics of patients included in and excluded from the propensity score–matched cohort

| **Variable** | **Included in matched cohort (n = 252)** | **Excluded due to missing covariates (n = 40)** | **p value** |
| --- | --- | --- | --- |
| Age, years | 70.6 ± 11.3 | 70.3 ± 11.9 | 0.83 |
| Female (%) | 77.9 | 77.5 | 1.00 |
| Disease duration, months | 85.0 [19.0–190.0] | 65.5 [13.6–125.3] | 0.06 |
| b/tsDMARD-naïve, n (%) | 33.6 | 35.0 | 0.78 |
| CRP, mg/dL [IQR] | 1.1 [0.1–4.0] | 0.3 [0.1–1.8] | 0.22 |
| CDAI | 20.3 ± 11.0 | 19.8 ± 11.1 | 0.76 |
| Glucocorticoid (%) | 47.7 | 57.5 | 0.24 |

Values are median [interquartile range] or mean (SD), unless otherwise indicated, b/tsDMARD: biologic or targeted synthetic disease-modifying antirheumatic drugs, CRP: C-reactive protein, CDAI: clinical disease activity index
